# Comparing the protective performances of 3 types of N95 filtering facepiece respirators during chest compressions: A randomized simulation study

**DOI:** 10.1097/MD.0000000000008308

**Published:** 2017-10-20

**Authors:** Hyungoo Shin, Jaehoon Oh, Tae Ho Lim, Hyunggoo Kang, Yeongtak Song, Sanghyun Lee

**Affiliations:** a Department of Emergency Medicine, College of Medicine; b Convergence Technology Center for Disaster Preparedness, Hanyang University; c Department of Emergency Medicine, College of Medicine, Hallym University, Seoul, Republic of Korea.

**Keywords:** airborne infection, chest compression, fit factor, N95 filtering facepiece respirator

## Abstract

**Objective:**

Healthcare providers in emergency departments should wear respirators for infection protection. However, the wearer's vigorous movements during cardiopulmonary resuscitation may affect the protective performance of the respirator. Herein, we aimed to assess the effects of chest compressions (CCs) on the protective performance of respirators.

**Methods:**

This crossover study evaluated 30 healthcare providers from 1 emergency department who performed CC with real-time feedback. The first, second, and third groups started with a cup-type, fold-type, and valve-type respirator, respectively, after which the respirators were randomized for each group. The fit factors were measured using a quantitative fit testing device before and during the CC in each experiment. The protection rate was defined as the proportion of respirators achieving a fit factor ≥100.

**Results:**

The fold-type respirator had a significantly greater protection rate at baseline (100.0% ± 0.0%) compared to the cup-type (73.6% ± 39.6%, *P* = .003) and valve-type respirators (87.5% ± 30.3%, *P* = .012). During the CC, the fit factor values significantly decreased for the cup-type (44.9% ± 42.8%, *P* < .001) and valve-type respirators (59.5% ± 41.7%, *P* = .002), but not for the fold-type respirator (93.2% ± 21.7%, *P* = .095).

**Conclusions:**

The protective performances of respirators may be influenced by CC. Healthcare providers should identify the respirator that provides the best fit for their intended tasks.

## 1 Introduction

Healthcare providers are at risk for being exposed to infectious diseases, especially in the emergency department, which often houses clustered and unspecified numbers of patients who may carry airborne and aerosolized infectious diseases. Thus, the American Centers for Disease Control and Prevention guidelines recommend extended use and limited reuse of N95 filtering facepiece respirators that are certified by the National Institute for Occupational Safety and Health (NIOSH).^[1]^ The protection that is provided by these devices is dependent on the filter's efficiency and seal quality, which is influenced by the shape of the sealing surface, the pressure generated by the tethering devices, the respiratory flow rate, and the wearer's movements.^[2]^ Thus, even if healthcare providers wear N95 filtering facepiece respirators, there is still a risk of infection related to their movements (e.g., during chest compressions).

Korean healthcare providers who wore N95 filtering facepiece respirators were reportedly infected by the Middle East Respiratory Syndrome coronavirus (MERS-CoV) after performing cardiopulmonary resuscitation (CPR) on an infected patient.^[3]^ Thus, N95 filtering facepiece respirators must be evaluated to confirm that they provide a sufficiently tight seal. This testing can be performed using qualitative or quantitative methods, and is recommended in the current guidelines for healthcare providers who need to wear N95 filtering facepiece respirators. The fit factor is a quantitative estimate of a respirator's fit for a specific individual, and typically estimates the ratio of substance concentrations in the ambient air versus inside the worn respirator.^[4]^ In this context, adequate protection is defined as the percentage of fit factor scores of ≥100, which indicates a good fit.^[5]^

The American Heart Association CPR guidelines recommend performing high-quality CPR to achieve optimal outcomes, which requires vigorous activity.^[6]^ However, to the best of our knowledge, no previous studies have evaluated the effects of movements during procedures that are performed in emergency medical centers (e.g., CCs) on respirator efficiency. Thus, we hypothesized that movements during CCs could influence the protection of N95 filtering facepiece respirators, and performed the present study to test this hypothesis.

## 2 Methods

### 2.1 Study design

This prospective randomized crossover simulation study was designed to identify differences in the protective performances of three N95 filtering facepiece respirators during CCs. The study was performed at the Hanyang University Simulation Center (Seoul, Republic of Korea) during August 2016. The local ethics committee approved this study in July 2016 (HYUH 2016–02–026–005), and the study's protocol was registered with the Clinical Research Information Service (cris.nih.go.kr: KCT0002012). The study was conducted in accordance with the Declaration of Helsinki (1964).

### 2.2 Participants

We recruited healthy 20- to 60-year-old healthcare providers from a tertiary medical center during August 2016. All participants were certified in basic life support by the American Heart Association. We excluded individuals with lung disease (uncontrolled chronic asthma or pneumonia), high blood pressure (systolic pressure of >160 mmHg or diastolic pressure of >95 mmHg), and wrist or lower back disease. The sample size was calculated based on a pilot study of 5 participants, which examined the fit factor before and during CCs. The mean fit factors were 199.74 (1.02) before the CC and 153.83 (60.85) during the CC. The estimated sample size calculation (G-power 3.1.2; Heine Heinrich University, Düsseldorf, German) revealed a required sample of 26 participants (effect size: 0.58, α-error: 0.05, power: 0.8); hence, 30 participants were enrolled to account for a 10% drop-out rate. All participants signed a written consent form before being included.

### 2.3 Equipment and material

**Figure 1:**
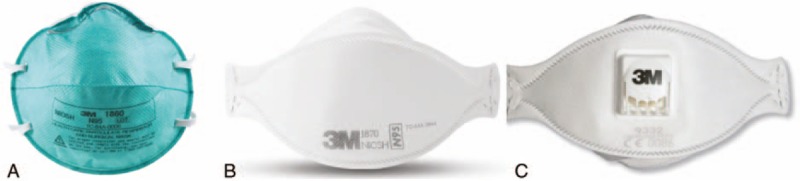
The quantitative fit test performed using the Porta-Count Plus device. Chest compressions were performed using a Resusci Anne Modular System Skill Reporter manikin on the Stryker ST104–747 bed. (A) The cup-type respirator is preformed into a cup shape (3 M 1860). (B) The fold-type respirator is flexible and free-folded (3 M 1870). (C) The valve-type respirator is similar to the fold-type and has a valve for reducing exhalation resistance (3 M 9332).

Three N95 or higher-level respirators were selected for this study (Fig. 1). The first was a cup-type respirator that is preformed into a cup shape (1860; 3 M, Elyria, OH). The second was a fold-type respirator that is flexible and 3-folded (1870; 3 M). The third was a valve-type respirator that is similar to the fold-type respirator and has a valve for reducing exhalation resistance (9332; 3 M). These respirators were used in emergency medical centers during the Korean MERS epidemic.

The quantitative fit of the respirators was tested using the PortaCount Plus 8038 device (TSI Inc., St. Paul, MN). This device is supported by a wire hanging around the wearer's neck, and is equipped with 1 sampling tube that is exposed to the atmosphere (measures ambient particles) and a second sampling tube that measures particles in the respirator. The fit factor was calculated by dividing the concentration of particles in ambient air (outside the respirator) by the concentration of particles inside the respirator. The fit factor values are reported based on a maximum score of 200, and a fit factor of >100 is defined as adequate protection.^[7]^

To reduce the impact of factors that may influence the fit factor, such as differences in the rate or depth of CCs, CCs were performed with real-time feedback using the Resusci Anne Modular System Skill Reporter manikin (9.89 kg; Laerdal Medical, Orpington, UK). Data regarding CPR quality were collected and monitored using VAM software (version 1.30.19 beta) and a laptop. The manikin was laid on a backboard (450 × 600 × 10 mm, 3 kg Lifeline Plastic; Sung Shim Medical Co., Bucheon, Korea) and placed on a bed (Transport Stretcher, 760 × 2110 mm, 228 kg; Stryker Co., Kalamazoo, MI). The height of the bed was adjusted to approximately the height of the participant's mid-thigh level.

### 2.4 Intervention

**Figure 2:**
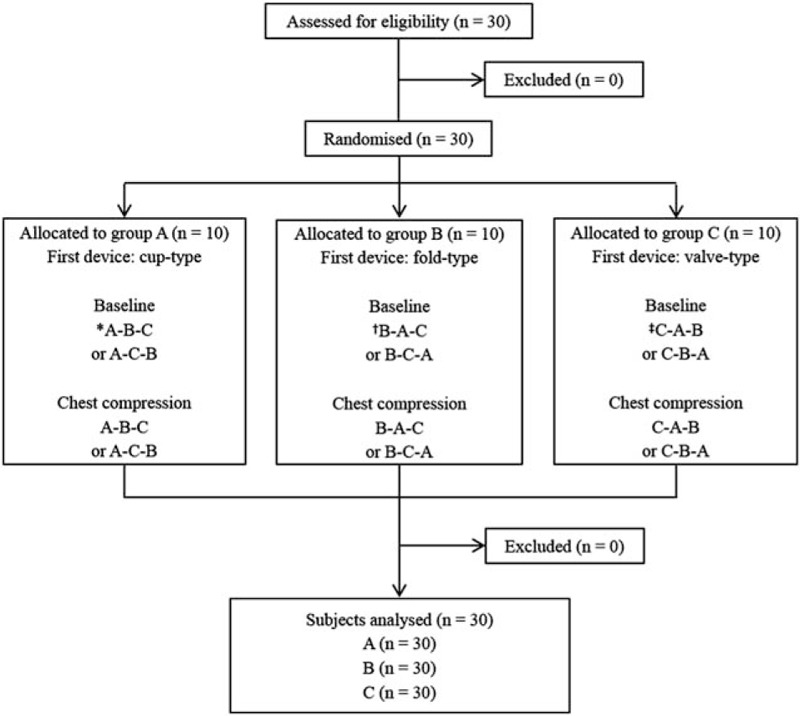
Flow chart of the study. A = cup-type respirator (3 M 1860), B = fold-type respirator (3 M 1870), C = valve-type respirator (3 M 9332).

All participants completed a brief questionnaire regarding their demographic information (age, sex, body weight, and height), experience using N95 filtering facepiece respirators, and experience performing CCs in clinical situations. The 30 enrolled participants were randomly allocated to 3 groups according to the first respirator type that they would use (*www.random.org*) (Fig. 2). All participants were prohibited from smoking, eating, chewing gum, and drinking (except plain water) for ≥30 minutes before starting the quantitative fit test, which was performed in a resuscitation room (24.3 m^3^) without an operating air conditioning system in an emergency medical center.

Sodium chloride aerosol (≥100 particles/cc) was generated using a TSI model 8026 particle generator. We performed a pretest using PortaCount Plus 8038 respirator fit tester to ensure that the ambient air contained enough particles for obtaining accurate fit factors.^[8]^ To minimize the effects of differences in the experience and training in wearing a respirator, which may influence the protective performance of respirators, we educated all participants regarding the respirators using manuals. Before starting the test, the participants were allowed to practice donning the respirators and to briefly review the department of infection management's instructions regarding wearing the respirators. The fit factors for all 3 respirators were measured at baseline (before the CCs) and during the CCs. Each participant performed 6 real-time quantitative mask fit tests. Before donning the respirator, the sampling tube was connected to a probe inside the respirator, without altering the respirator's shape.

During the baseline phase, the participants wore the respirators and the fit factors were measured during 2 minutes of normal breathing after the participant checked the seal in accordance with the manufacturer's instructions. During the CCs, the participants wore the respirators before starting the CCs, and began performing compressions using the manikins on the beds according to the 2015 American Heart Association Basic Life Support guidelines. All participants were provided a 10-minute break between each test.

### 2.5 Primary and secondary outcomes

The primary outcome was the adequate protection rate, which was calculated as the proportions of respirators achieving a fit factor of ≥100 (indicating a good fit). We also measured the real-time fit factors for the three respirators at baseline and during the CCs, and asked the participants to report their preferred respirator for use in clinical situations.

### 2.6 Statistical analysis

All data were compiled using Microsoft Excel (Microsoft Corp., Redmond, WA) and analyzed using IBM SPSS software (version 20.0; IBM Corp., Armonk, NY). Categorical data were reported as the absolute numbers and percentages, and continuous data were reported as the median values and interquartile ranges because the data were not normally distributed. The adequate protection rates for the 3 respirators were compared using the Friedman test (nonparametric data) or repeated-measures analysis of variance (continuous parametric data). A post-hoc analysis was performed using the Wilcoxon rank-sum test (nonparametric data) or the paired *t* test using Bonferroni correction (parametric data). For all analyses, differences with a *P* value of <.05 were considered statistically significant.

## 3 Results

### 3.1 Protective performance at baseline

All 30 participants completed the study; their general characteristics are shown in Table 1. First, we measured the adequate protection rates (proportion of respirators achieving a fit factor of ≥100); the results are shown in Table 2. The baseline adequate protection rates were significantly different for the 3 respirators (*P* = .002). The fold-type respirator provided the greatest adequate protection rate (100.0% ± 0.0%), followed by the valve-type (87.5% ± 30.3%) and cup-type respirators (73.7% ± 39.6%).

**Table 1:**
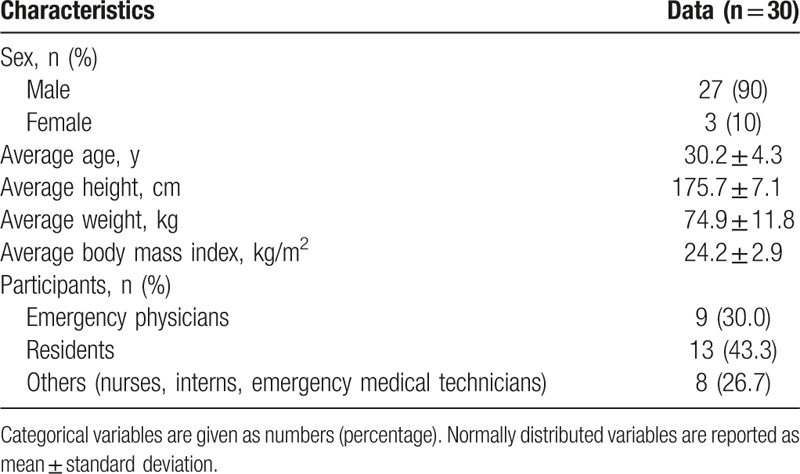
General characteristics.

**Table 2:**
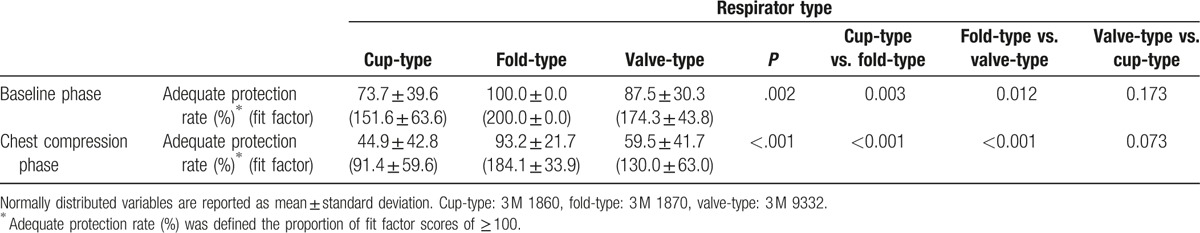
Comparison the adequate protection (fit factor) rate during the baseline and chest compression phases for three N95 respirators.

### 3.2 Protective performance during CCs

**Figure 3:**
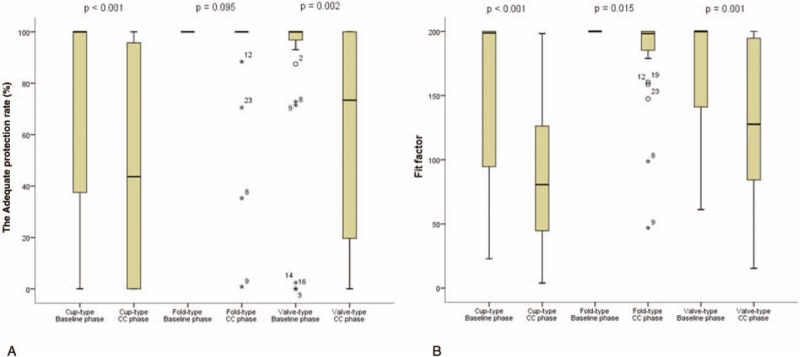
The Adequate protection rate (A) and fit factor (B) for the 3 respirators during the baseline and chest compression phases. The protective performance of the fold-type respirator did not decrease during the chest compressions. The adequate protection rates and fit factors of the cup-type and valve-type respirators decreased during the chest compressions. Cup-type: 3 M 1860, fold-type: 3 M 1870, valve-type: 3 M 9332.

The mean adequate protection rate decreased during the CCs for all 3 respirators (Table 2). However, there were no significant differences in the quality of CCs for the 3 respirators (adequate compression depth: 97.6% ± 4.4%, *P* = .95; adequate compression rate: 96.0% ± 5.2%, *P* = .198; adequate position: 97.4% ± 4.5%, *P* = .524; complete recoil: 99.0% ± 3.4%, *P* = .651). The cup-type and valve-type respirators provided lower adequate protection rates during the CCs than at baseline (cup-type: *P* < .001, valve-type: *P* = .002). There was no significant difference between the baseline and CC values for the fold-type respirator (*P* = .095), which provided the highest adequate protection rate (Fig. 3).

### 3.3 Respirator preferences

Twenty-eight participants (93.3%) preferred the fold-type or valve-type respirators, and only 2 participants (6.7%) preferred the cup-type respirator.

## 4 Discussion

Infection prevention and control remain major challenges for the emergency medical system, as emergency departments have complex and dynamic environments. Acutely ill patients may spread infectious diseases to healthcare personnel in the emergency department, and these personnel may subsequently transmit the infection to other patients during their treatment.^[9]^ Thus, healthcare providers should wear NIOSH-certified N95 or greater level respirators to protect against airborne droplets, based on the Centers for Disease Control and Prevention guidelines.^[1]^ Securing respiratory protection may also be important for the health care provider during CCs. To our knowledge, no previous studies have examined the protective performances of respirators during CC in the emergency room. Accordingly, we performed this study to assess the influence of movement during CC on the protective performance of three N95 filtering facepiece respirators. The movement during CC decreased the acceptable performance rate of the cup-type and valve-type, but not the fold-type respirators.

If a respirator's fit is compromised, leakage can occur through various routes, including the filter, face-seal leakage, and the exhalation valve.^[10]^ However, all filters in this study were N95 or higher. Moreover, face seal leakage is a main component of respirator leakage.^[11]^ Thus, the movement of the wearer may influence the protective performance of N95 filtering facepiece respirators. To reflect the effect of movements, simulated workplace testing for respirator fit typically considers 8 standard exercises: normal breathing, deep breathing, turning the head side to side, moving the head up and down, talking, grimacing, bending over, and normal breathing.^[12,13]^ It is likely that the movement during CC differs from that during these 8 standard exercises. Our simulation study revealed that dynamic motions, like those during CC, decreased the protective performance of the respirators. Although CC using mechanical devices could solve this problem, manual CCs remain the standard of care for the treatment of cardiac arrest.^[14]^ Thus, we believe that the user should consider the required motions during CC when selecting and fitting a respirator for procedures that involve a risk of infection.

The physical characteristics of respirators could have different effects on the protective performance during CC. In this context, the fold-type and valve-type respirators have flexible sealing surfaces, whereas the cup-type does not, suggesting that users may more easily customize the relative shape of the fold-type and valve-type respirators to achieve a better face seal. The low baseline fit factor and high reduction in the adequate performance rate for the cup-type respirator may be related to the fixed shape of the cup, as the fit factor can be affected by the wearer's size and shape. In the present study, only 2 participants reported preferring the cup-type respirator, which may be related to the mask's rigidity causing facial discomfort. The valve-type respirator may have a better breathing performance, as the valve can help to quickly transfer the exhaled gas to the outside of the respirator. Although the valve in a respirator reduces exhalation resistance,^[10]^ it may also increase the risk of leakage, and additional studies are needed to quantify the leakage through the exhalation valve. Furthermore, although the fold-type respirator showed better performance than the valve-type respirator in our study, its wearing comfort may be suboptimal, and a future study of the contact characteristics of these masks is warranted.^[15,16]^

The skill level of the operator and training in wearing a respirator could affect the protective performance of respirators. To reduce the impact of the degree of training and experience on the respirator fit, all participants received real-time feedback after their respirator training, based on the manufacturer's instructions. Such a training course may help improve the baseline respirator fit for all types of respirators. To reduce the risk of infection, it is important to ensure that the wearer knows how to properly fit the respirator before entering an area of potential infectious spread.^[17]^

Based on the present study, it appears that the respirator shape and wearer movements can affect the respirator function, and fit testing is needed to confirm whether the respirator is functioning properly during tasks that involve moving the head (e.g., CCs). This type of testing may help improve the safety and protection of healthcare providers in emergency departments. Furthermore, healthcare providers should select and fit appropriate respirators based on the manufacturer's instructions.

There were several limitations in this study. First, we evaluated only 3 types of respirators that were used during the South Korean MERS outbreak (the cup-type and fold-type respirators had an N95 rating, and the valve-type respirator had an FFP3 rating). Therefore, clinical trials with other respirator types are needed to confirm the effects of movements during CCs. Second, we used a manikin during the CCs, and it is possible that the participants approached this exercise in a different manner compared to their approach during an actual cardiac arrest. Third, we did not consider the effects of numerous other tasks that may be involved in CPR, such as defibrillation, preparation for intubation, and intravenous drug administration. Simultaneously performing these tasks might influence the actual CCs in a human case. Fourth, the participants were mainly young men, and it is possible that women or older individuals might have different CC performances. Fifth, we evaluated relatively experienced healthcare providers (certified in basic life support by the American Heart Association), and different results might have been observed if we had recruited less experienced participants.

## 5 Conclusions

The results of the present study indicate that the protective performance of N95 filtering facepiece respirators is affected by the wearer's movements. Thus, healthcare providers should be educated to properly select, fit, and wear appropriate N95 filtering facepiece respirators in the emergency department.

## References

[1] Tablan OC Anderson LJ Besser R CDC; Healthcare Infection Control Practices Advisory Committee. Guidelines for preventing health-care-associated pneumonia, 2003: recommendations of CDC and the Healthcare Infection Control Practices Advisory Committee. MMWR Recomm Rep 2004;53:1–36. 15048056

[2] Kolesar ES Cosgrove DJ De La Barre CM Comparison of respirator protection factors measured by two quantitative fit test methods. Aviat Space Environ Med 1982;53:1116–22. 7150173

[3] The Korea Times. Nurse awarded for fight against MERS. Available at: https://www.koreatimes.co.kr/www/news/people/2016/09/178_211072.html; 2016. Accessed December 3, 2016.

[4] United States Department of Labor. Occupational Safety and Health Administration. Available at: https://www.osha.gov/pls/oshaweb/owadisp.show_document?p_table=STANDARDS&p_id=12716. Accessed December 5, 2016.

[5] Bergman M Zhuang Z Brochu E Fit assessment of N95 filtering-facepiece respirators in the U.S. Centers for Disease Control and Prevention Strategic National Stockpile. J Int Soc Respir Prot 2015;32:50–64. 26877587 PMC4752193

[6] Berg RA Hemphill R Abella BS Part 5: adult basic life support. 2010 American Heart Association Guidelines for Cardiopulmonary Resuscitation and Emergency Cardiovascular Care. Circulation 2010;122 (18 suppl 3):S685–705. 20956221 10.1161/CIRCULATIONAHA.110.970939

[7] Niezgoda G Kim JH Roberge RJ Flat fold and cup-shaped N95 filtering facepiece respirator face seal area and pressure determinations: a stereophotogrammetry study. J Occup Environ Hyg 2013;10:419–24. 23767820 10.1080/15459624.2013.801246 PMC4545596

[8] Lawrence RB Duling MG Calvert CA Comparison of performance of three different types of respiratory protection devices. J Occup Environ Hyg 2006;3:465–74. 16857645 10.1080/15459620600829211

[9] Liang SY Theodoro DL Schuur JD Infection prevention in the emergency department. Ann Emerg Med 2014;64:299–313. 24721718 10.1016/j.annemergmed.2014.02.024 PMC4143473

[10] Rengasamy S Eimer BC Szalajda J A quantitative assessment of the total inward leakage of NaCl aerosol representing submicron-size bioaerosol through N95 filtering facepiece respirators and surgical masks. J Occup Environ Hyg 2014;11:388–96. 24275016 10.1080/15459624.2013.866715 PMC4589201

[11] Grinshpun SA Haruta H Eninger RM Performance of an N95 filtering facepiece particulate respirator and a surgical mask during human breathing: two pathways for particle penetration. J Occup Environ Hyg 2009;10:593–603. 10.1080/15459620903120086 PMC7196699 19598054

[12] Lei Z Ji X Li N Simulated effects of head movement on contact pressures between headforms and N95 filtering facepiece respirators part 2: simulation. Ann Occup Hyg 2014;58:1186–99. 25187035 10.1093/annhyg/meu064

[13] Duling MG Lawrence RB Slaven JE Simulated workplace protection factors for half-facepiece respiratory protective devices. J Occup Environ Hyg 2007;4:420–31. 17474032 10.1080/15459620701346925

[14] Brooks SC Anderson ML Bruder E Part 6: alternative techniques and ancillary devices for cardiopulmonary resuscitation. 2015 American Heart Association Guidelines Update for Cardiopulmonary Resuscitation and Emergency Cardiovascular Care. Circulation 2015;132 (18 suppl 2):S436–43. 26472994 10.1161/CIR.0000000000000260

[15] Cai M Shen S Li H Study of contact characteristics between a respirator and a headform. J Occup Environ Hyg 2016;13:D50–60. 26558322 10.1080/15459624.2015.1116699

[16] Cai M Shen S Li H The effect of facial expressions on respirators fit. Comput Methods Biomech Biomed Engin 2017;20:1122–31. 28580847 10.1080/10255842.2017.1336549

[17] Han DH Correlation of fit factors for respirators and anthropometric dimension. Korean J Prev Med 1998;31:440–8.

